# Clinical outcomes of total hip arthroplasty after femoral neck fractures vs. osteoarthritis at one year follow up—A comparative, retrospective study

**DOI:** 10.1007/s00264-024-06242-0

**Published:** 2024-07-06

**Authors:** Bogdan Obada, Vlad Georgeanu, Madalina Iliescu, Andrei Popescu, Lucian Petcu, Dan Ovidiu Costea

**Affiliations:** 1 Orthopaedic Traumatology Clinic, Ovidius” University of Constanta, Constanta, Romania; 2 Orthopaedic Traumatology Clinic, UMF “Carol Davila, Bucharest, Romania; 3 Medical Rehabilitation Clinic, Ovidius” University of Constanta, Constanta, Romania; 4 Romanian Shoulder Institute, Bucharest, Romania; 5 Ovidius” University of Constanta, Constanta, Romania; 6 General Surgery Clinic, Ovidius” University of Constanta, Constanta, Romania

**Keywords:** Total hip arthroplasty, Osteoarthritis, Femoral neck fractures, Outcomes

## Abstract

**Purpose:**

The objective of the study is to determine if there was a difference in medical complications and in-hospital mortality among the patients who underwent THA for femoral neck fracture relative to same procedure for elective patients with coxarthrosis.

**Methods:**

We compared characteristics and short-term outcomes during the rehabilitative postsurgical period. We included all patients older than 45 years who underwent THA for primary/secondary hip arthritis and displaced femoral neck fractures type Garden III and IV. Clinical examination, functional outcome and radiographic evaluation were performed during follow-up. Patients were evaluated at the following time points: preoperatively, postoperatively at three days, six weeks, 12 weeks and one year and we registered Visual Analogue Scale (VAS) pain score, Harris Hip Score (HHS), the Western Ontario McMaster Osteoarthritis Index (WOMAC), internal and external rotation of the hip and operated limb length compared with the opposite.

**Results:**

There is no significant statistically differences between the two groups regarding the preoperative comorbidities. The frequencies of patients experiencing in-hospital and 30-day postoperative complications were generally low and same in groups we studied. The mean quantity of surgical blood loos during the operation was significantly higher in the hip fracture group compared with elective patient group with OA (340.09 ± 86.03 vs 309.43 ± 102.52). With respect to postoperative recovery the patients with THA after FNF were mobilized by active walking a little bit faster as the patient with OA (2.77 ± 1.18 days vs 3.1 ± 1.14 days). The average inpatient hospital length of stay after THA for OA was 11.07 days compared to 13.41 days following a THA for FNF.

**Conclusion:**

Our study showed that THA for treatment of an acute fracture of the femoral neck in an elderly patient can provide results comparable to those of patients who received THA for OA and we found that the results are similar.

## Introduction

Arthroplasty of the hip is generally recommended in displaced femoral neck fractures due to poor healing and risk for avascular necrosis after osteosynthesis and is also a standard surgical procedure indicated for the patients with severe osteoarthritis (OA).

Total hip arthroplasty (THA) is a surgery which can improve mobility and pain but is also associated with important postoperative complications. Patients undergoing hip replacement for a femoral neck fracture (FNF) are at substantially higher risk of medical complications including mortality compared with elective patients undergoing THA for coxarthrosis probably due to advanced age and comorbidities of hip fracture patients. There are few studies who directly compared the postoperative results after THA for the patients with osteoarthritis and for the patients with FNF. They also suggested that patients with FNF are more likely to experience postoperative complications. [1–3]

The choice of surgical approach in hip arthroplasty is an important part of preoperative planning. The aspects considered are soft tissue protection, good access to proximal femur and acetabulum, stability of the prosthesis, postoperative pain, and rapid recovery of the function. Lateral approach is proved to be associated with better prosthetic stability compared with other approaches. The FNF patients are older and weaker than the coxarthrosis patients and with an altered mental status. The prefracture range of motion is better preserved than OA patients which have limited motion, a fragile bone quality and that lead to higher risk of dislocation for the patients with FNF after THA. Our surgeon’s preference was a modified lateral approach of Hardinge. [4, 5]

The objective of the study is to determine if there was a difference in medical complications and in-hospital mortality among the patients who underwent THA for femoral neck fracture relative to same procedure for elective patients with coxarthrosis. We compared characteristics and short-term outcomes during the rehabilitative postsurgical period.

## Material and methods

This study was approved by the ethical committee of Emergency Clinical County Hospital “St Apostle Andrei” of Constanta, Romania and written informed consent was obtained from all the patients. We retrospectively reviewed the records of 864 consecutive patients with THA performed by six senior orthopaedic surgeons between January 2017 and December 2021 in the Orthopaedics and Traumatology Clinic.

We included all patients older than 45 years who underwent THA for primary/secondary hip arthritis and displaced femoral neck fractures type Garden III and IV. Exclusion criteria were patients with multiple trauma, femoral neck fractures type I or II according to Garden classification, arthritis after acetabular fractures, bilateral hip arthroplasty, hip revision arthroplasty, prior hip surgery, presence of infection, neuromuscular disease, dementia or other cognitive disorders. We did not exclude any patient based on body mass index.

The data collected for analysis were age, gender, associated pathology, diagnosis, prosthesis type, duration of hospitalization and postoperative complications (local haematoma, early and delayed periprosthetic joint infection, dislocations). We also recorded characteristics related to the surgery: incision length, surgery time, blood loss, postoperative transfusion needs and intraoperative complications (fractures, neurological and vascular lesions).

All THAs were performed using a modified lateral approach (Hardinge) with the patient in supine position. Cemented or uncemented (depending on age and bone condition) prosthesis were used. As cemented implant we used Zimmer Biomet ZCA App-Poly acetabular cup with 32 mm CoCr head, Taperloc hip femoral stem and as uncemented implant we used Zimmer Trilogy acetabular system, 32 mm liner Longevity crosslinked polyethylene with 32 mm CoCr head, Taperloc porous coated stem.

Perioperatively, most of the patients received the same standard intravenous antibiotic prophylaxis. All the patients received prophylaxis of thromboembolism (enoxaparin sodium) for six weeks. Drainage was used for all the cases for 24 h. for all the operations. Blood management included administration of blood products or allogenic transfusion. For cemented prosthesis, antibiotic loaded cement was used.

Physical therapy started progressively on day day postoperative with partial weight-bearing as tolerate using a walker. Clinical examination, functional outcome and radiographic evaluation were performed during follow-up. Patients were evaluated at the following time points: preoperatively, postoperatively at three days, six weeks, 12 weeks and one year and we registered Visual Analogue Scale (VAS) pain score, Harris Hip Score (HHS), the Western Ontario McMaster Osteoarthritis Index (WOMAC), internal and external rotation of the hip and operated limb length compared with the opposite. Conventional antero-posterior pelvis and cross table lateral radiographic projections were obtained preoperative, postoperative, at six weeks and at 12 months.

The hip range of motion was determined in the standard manner using an universal goniometer. Special attention was paid to internal/external rotation that were measured with the patient in the seated position, with the hip and knee flexed 90°. These hip motions, especially in the extremes are traditionally related to hip dislocation in the early postoperative period, so is very important to gain them without additional risks.

### Statistical analysis

The statistical analysis was performed using IBM SPSS statistics software version 25. Data are presented as mean ± standard deviation (SD) for continuous variables, or as percentages for categorical variables. An ANOVA Test with repeated measures and a Paired Sample Test were used to see changes in the intervention. The normality of the test variables was estimated with Kolmogorov–Smirnov Tests of Normality. For ANOVA Test with repeated measures, Sphericity was tested with Mauchly’s test. If sphericity is violated (p < 0.05), the Greenhouse–Geisser, Huynh–Feldt and lower bound methods are used to correct the within-subjects tests. Post hoc analysis with a Bonferroni adjustment for multiple comparisons was used to discover which specific mean values differed. For categorical variables, the z test was used to compare proportions between the two groups. The significance level α was set at 0.05.

## Results

In our study population, 526 patients underwent elective THA for coxarthrosis, and 338 patients received THR for FNF. In the same period, the group of elective patients were larger than the hip fracture group. The female to male ratio was higher in the FNF group probably due to osteoporosis which is more frequent to women. Regarding the aetiology of coxarthrosis, most of the cases was primary (idiopathic), followed by secondary coxarthrosis due to rheumatoid diseases which not influenced in any way the results of our study.

There is no significant statistically differences between the two groups regarding the preoperative comorbidities. The frequencies of patients experiencing in-hospital and 30-day postoperative complications were generally low and same in groups we studied.

**Fig. 1:**
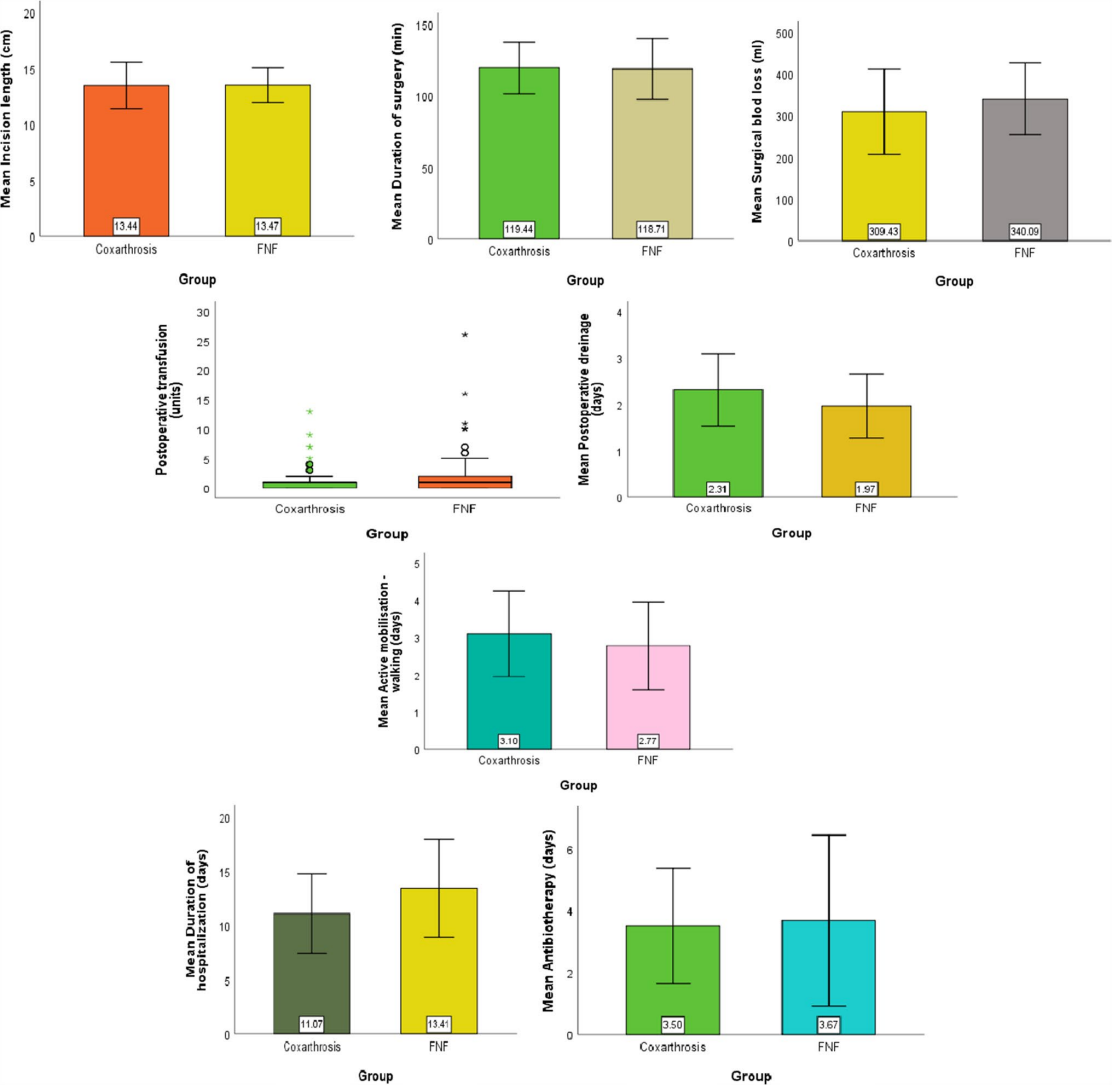
Characteristics of the surgical procedure

The mean quantity of surgical blood loos during the operation was significantly higher in the hip fracture group compared with elective patient group with OA (340.09 ± 86.03 vs 309.43 ± 102.52). With respect to postoperative recovery the patients with THA after FNF were mobilized by active walking a little bit faster as the patient with OA (2.77 ± 1.18 days vs 3.1 ± 1.14 days). The average inpatient hospital length of stay after THA for OA was 11.07 days compared to 13.41 days following a THA for FNF (Tables 1, 2, 3) (Fig. 1).

**Table 1:** Demographic data

	Coxarthrosis (n = 526)		FNF (n = 338)		z*	p
	n1	%	n2	**%**		
**Gender**						
Men	275	52.28	127	37.57	4.230	< 0.001
Women	251	47.72	211	62.43	-4.230	< 0.001
**Prosthesis type**						
Cemented	226	42.97	244	72.19	-8.417	< 0.001
Noncemented	300	57.03	94	27.81	8.417	< 0.001
**Associated pathology**						
Diabetes	67	12.74	43	12.72	0.007	0.995
High blood pressure	336	63.88	215	63.61	0.080	0.936
Obesity	92	17.49	35	10.36	2.891	0.004
Heart disease	197	37.45	157	46.45	-2.624	0.009
**Complications**						
Acetabulum/femur fracture	2	0.38	0	0.00	1.135	0.256
Dislocation of prosthesis	6	1.14	4	1.18	-0.057	0.954
Deep infection	7	1.33	5	1.48	-0.182	0.856
Deep haematoma	73	13.88	40	11.83	0.870	0.385
Thrombophlebitis	2	0.38	1	0.30	0.206	0.837
Pulmonary thromboembolism	3	0.57	1	0.30	0.580	0.562
Periprosthetic fracture	3	0.57	5	1.48	-1.361	0.173
Heterotopic ossification	6	1.14	5	1.48	-0.433	0.665
Prosthesis loosening	3	0.57	0	0.00	1.391	0.164
Limb shortening	9	1.71	8	2.37	-0.677	0.498

* z test to compare proportions

If p < 0.05 it is considered that are difference between the compared series

**Table 2:** Data related to the surgery

	Group		Mean	SD	Min	Max	Percentiles		
		N					P25	Median	P75
Incision length (cm)	Coxarthrosis	526	13.44	2.08	10	20	12.00	13.00	14.25
	FNF	338	13.47	1.56	10	18	12.00	13.00	15.00
Duration of surgery (min)	Coxarthrosis	526	119.44	17.88	90	170	110.00	120.00	130.00
	FNF	338	118.71	21.45	80	160	100.00	120.00	140.00
Surgical blod loss (ml)	Coxarthrosis	526	309.43	102.52	120	770	210.00	300.00	400.00
	FNF	338	340.09	86.03	200	700	300.00	350.00	400.00
Postoperative transfusion (units)	Coxarthrosis	526	0.87	1.16	0	13	0	1.00	1.00
	FNF	338	1.54	2.32	0	26	0	1.00	2.00
Postoperative dreinage (days)	Coxarthrosis	526	2.31	0.78	1	8	2.00	2.00	3.00
	FNF	338	1.97	0.69	1	5	2.00	2.00	2.00
Active mobilisation—walking (days)	Coxarthrosis	526	3.10	1.14	1	12	2.00	3.00	4.00
	FNF	338	2.77	1.18	1	13	2.00	3.00	3.00
Duration of hospitalization (days)	Coxarthrosis	526	11.07	3.67	4	33	9.00	10.00	13.00
	FNF	338	13.41	4.50	3	42	10.00	13.00	15.00
Antibiotherapy (days)	Coxarthrosis	526	3.50	1.86	2	30	3.00	3.00	4.00
	FNF	338	3.67	2.76	2	37	3.00	3.00	4.00

**Table 3:** t-test for Equality of Means Coxarthrosis vs. FNF

	t	df	p	Mean Difference	95% CI of the Difference	
					Lower	Upper
Incision length (cm)	-0.237	842.089	0.813	-0.029	-0.273	0.214
Duration of surgery (min)	0.515	625.131	0.607	0.722	-2.034	3.478
Surgical blod loss (ml)	-4.737	803.267	< 0.001	-30.655	-43.358	-17.953
Postoperative dreinage (days)	6.813	783.714	< 0.001	0.344	0.245	0.444
Active mobilisation—walking (days)	4.056	862	< 0.001	0.327	0.169	0.485
Duration of hospitalization (days)	-7.988	614.264	< 0.001	-2.337	-2.912	-1.763
Antibiotherapy (days)	-1.000	532.739	0.318	-0.171	-0.506	0.165

If p < 0.05 it is considered that are difference between the compared series

***For coxarthrosis group***, a repeated measures ANOVA with a Huynh–Feldt correction determined that mean Harris score differed statistically significantly between time points (*F*(1.844, 967.869) = 12,546.423, *P* < 0.001). Post hoc analysis with a Bonferroni adjustment revealed that Harris score was statistically significantly increased from 6 to 12 weeks (-9.605 (95% CI, -9.825 to -9.384), *p* < 0.001), and from 12 weeks to 1 year (-6.010 (95% CI, -6.230 to -5.789), *p* < 0.001).

A repeated measures ANOVA with a Huynh–Feldt correction determined that mean Womac score differed statistically significantly between time points (*F*(1.765, 926.850) = 6247.813, *P* < 0.001). Post hoc analysis with a Bonferroni adjustment revealed that Womac score was statistically significantly decreased from six to 12 weeks (2.622 (95% CI, 2.529 to 2.714), *p* < 0.001), and from 12 weeks to one year (2.492 (95% CI, 2.368 to 2.599), *p* < 0.001).

A Paired Samples Test determined that mean VAS score differed statistically significantly between time points (*t* = 51.526*, df* = 525, *p* < 0.001). The test revealed that VAS score was statistically significantly decreased from three days to six weeks (2.122 (95% CI, 2.041 to 2.203),* p* < 0.001).

A Paired Samples Test determined that mean ROM Internal rotation score differed statistically significantly between time points (*t* = -122.878*, df* = 525, *p* < 0.001). The test revealed that ROM Internal rotation score was statistically significantly increased from six to 12 weeks (-14.698 (95% CI, -14.933 to -14.463),* p* < 0.001). A Paired Samples Test determined that mean ROM External rotation score differed statistically significantly between time points (*t* = -102.011*, df* = 525, *p* < 0.001). The test revealed that ROM External rotation score was statistically significantly increased from six to 12 weeks (-14.947 (95% CI, -15.235 to -14.659),* p* < 0.001).

***For FNF group***, a repeated measures ANOVA with a Huynh–Feldt correction determined that mean Harris score differed statistically significantly between time points (*F*(1.541, 519.327) = 5630.846, *p* < 0.001). Post hoc analysis with a Bonferroni adjustment revealed that Harris score was statistically significantly increased from six weeks to 12 weaks (-9.689 (95% CI, -10.029 to -9.350), *p* < 0.001), and from 12 weeks to one year (-6.080 (95% CI, -6.357 to -5.803), *p* < 0.001).

A repeated measures ANOVA with a Huynh–Feldt correction determined that mean Womac score differed statistically significantly between time points (*F*(1.580, 532.507) = 5433.431, *p* < 0.001). Post hoc analysis with a Bonferroni adjustment revealed that Harris score was statistically significantly decreased from six weeks to 12 weaks (2.216 (95% CI, 2.130 to 2.302), *p* < 0.001), and from 12 weeks to 1 year (2.364 (95% CI, 2.268 to 2.460), *p* < 0.001).

A Paired Samples Test determined that mean VAS score differed statistically significantly between time points (*t* = 33.319*, df* = 337, *p* < 0.001). The test revealed that VAS score was statistically significantly decreased from three days to six weeks (2.852 (95% CI, 2.684 to 3.020),* p* < 0.001).

**Fig. 2:**
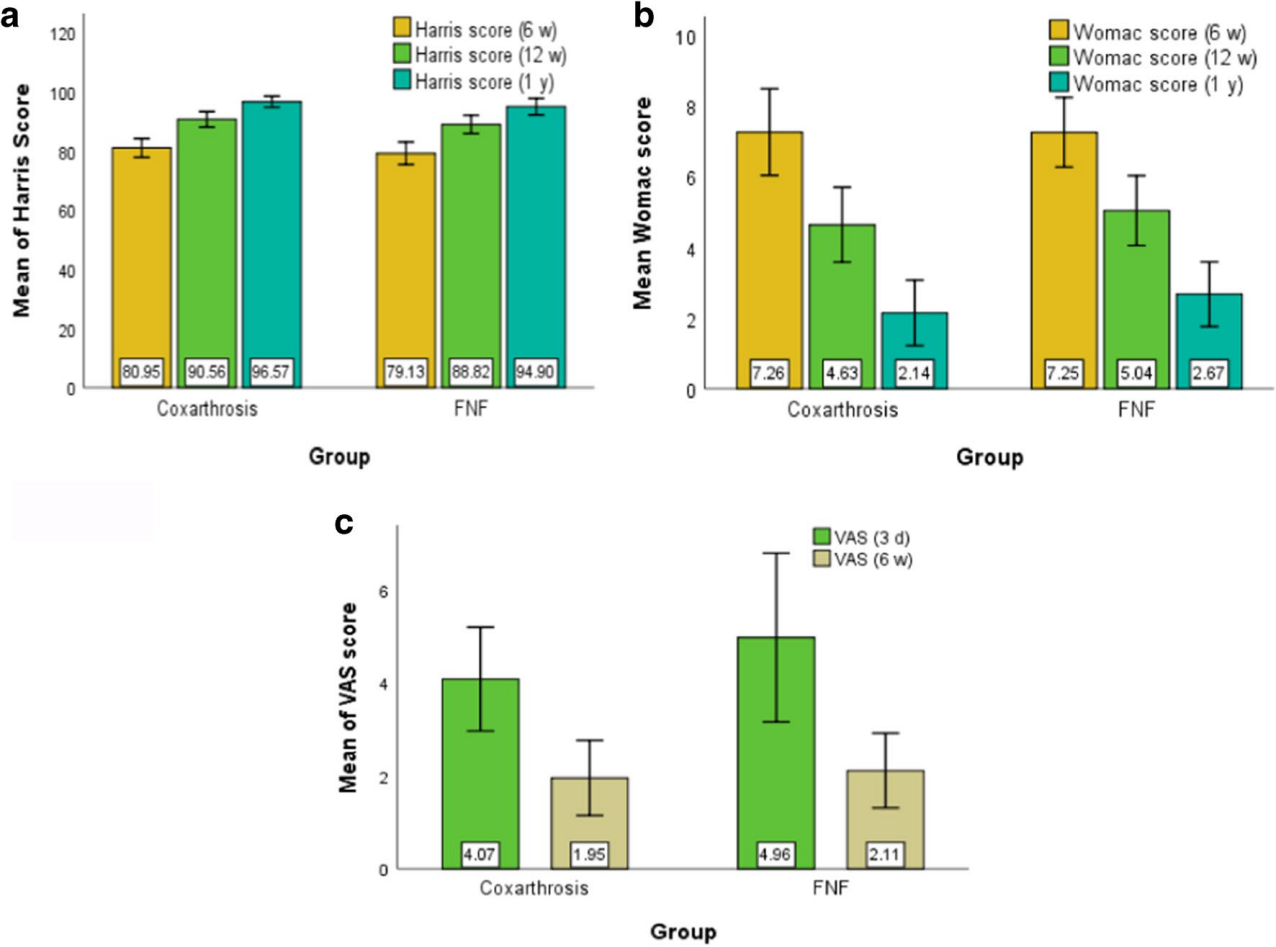
**a** Harris mean Score. **b** Womac mean Score **c**. VAS mean Score

**Fig. 3:**
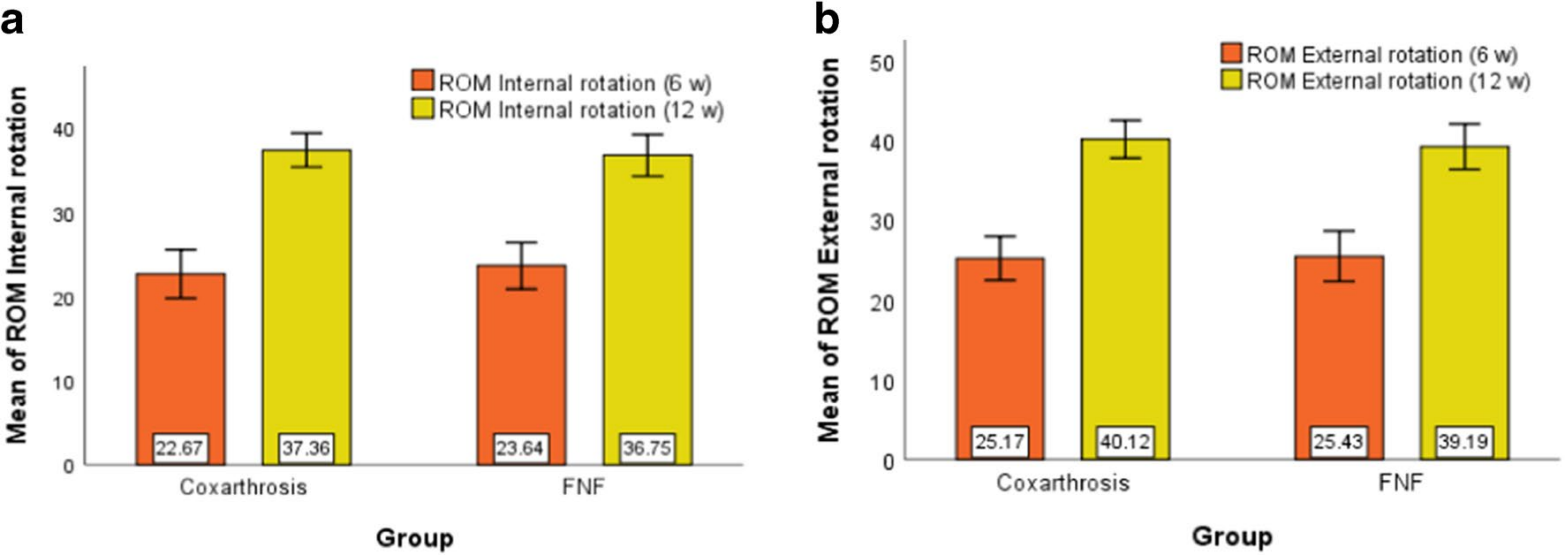
**a** ROM Internal Rotation mean Score **b** ROM External Rotation mean Score

A Paired Samples Test determined that mean ROM Internal rotation score differed statistically significantly between time points (*t* = -120.922*, df* = 337, *p* < 0.001). The test revealed that ROM Internal rotation score was statistically significantly increased from six to 12 weeks (-13.107 (95% CI, -13.320 to -12.893),* p* < 0.001). A Paired Samples Test determined that mean ROM External rotation score differed statistically significantly between time points (*t* = -94.094*, df* = 337, *p* < 0.001). The test revealed that ROM External rotation score was statistically significantly increased from six to 12 weeks (-13.763 (95% CI, -14.051 to -13.476),* p* < 0.001) (Tables 4, 5) (Figs. 2, 3).

**Table 4:** Functional evaluation

	Group		Mean	SD	Min	Max	Percentiles		
		N					P25	Median	P75
Harris score (6 w)	Coxarthrosis	526	80.95	3.177	70	91	79.75	81.00	83.00
	FNF	338	79.13	3.812	66	90	77.00	80.00	82.00
Harris score (12 w)	Coxarthrosis	526	90.56	2.627	80	97	89.00	90.00	92.00
	FNF	338	88.82	3.083	70	95	87.00	89.00	91.00
Harris score (1 y)	Coxarthrosis	526	96.57	1.86	90	100	95.00	97.00	98.00
	FNF	338	94.90	2.84	83	100	93.00	95.00	97.00
Womac score (6 w)	Coxarthrosis	526	7.26	1.23	4	10	7.00	7.00	8.00
	FNF	338	7.25	0.99	5	9	7.00	7.00	8.00
Womac score (12 w)	Coxarthrosis	526	4.63	1.06	1	8	4.00	5.00	5.00
	FNF	338	5.04	0.99	3	8	4.00	5.00	6.00
Womac score (1 y)	Coxarthrosis	526	2.14	0.92	1	6	2.00	2.00	3.00
	FNF	338	2.67	0.91	1	5	2.00	3.00	3.00
VAS (3 d)	Coxarthrosis	526	4.07	1.11	1	7	3.00	4.00	5.00
	FNF	338	4.96	1.81	2	9	4.00	4.00	6.00
VAS (6 w)	Coxarthrosis	526	1.95	0.81	1	6	1.00	2.00	2.00
	FNF	338	2.11	0.80	1	5	2.00	2.00	3.00
ROM Internal rotation (6 w)	Coxarthrosis	526	22.67	2.86	14	34	21.00	22.00	25.00
	FNF	338	23.64	2.75	17	30	22.00	24.00	25.00
ROM Internal rotation (12 w)	Coxarthrosis	526	37.36	2.01	30	49	36.00	37.00	39.00
	FNF	338	36.75	2.48	30	42	35.00	37.00	39.00
ROM External rotation (6 w)	Coxarthrosis	526	25.17	2.78	19	35	24.00	25.00	27.00
	FNF	338	25.43	3.19	4	40	23.00	25.00	28.00
ROM External rotation (12 w)	Coxarthrosis	526	40.12	2.36	33	45	39.00	40.00	41.00
	FNF	338	39.19	2.85	30	45	37.00	39.00	41.00

**Table 5:** T-test for Equality of Means Coxarthrosis vs. FNF

	t	df	p	Mean Difference	95% Confidence Interval of the Difference	
					Lower	Upper
Harris score (6 w)	7.316	625.072	< 0.001	1.824	1.335	2.314
Harris score (12 w)	8.564	635.930	< 0.001	1.739	1.341	2.138
Harris score (1 y)	9.581	523.180	< 0.001	1.669	1.327	2.011
Womac score (6 w)	0.068	819.647	0.946	0.005	-0.143	0.154
Womac score (12 w)	-5.657	755.883	< 0.001	-0.401	-0.540	-0.262
Womac score (1 y)	-8.293	727.232	< 0.001	-0.529	-0.654	-0.404
VAS (3 d)	-8.094	501.518	< 0.001	-0.889	-1.105	-0.673
VAS (6 w)	-2.829	862	0.005	-0.159	-0.269	-0.049
ROM Internal rotation (6 w)	-4.959	862	< 0.001	-0.974	-1.359	-0.588
ROM Internal rotation (12 w)	3.834	612.162	< 0.001	0.618	0.301	0.934
ROM External rotation (6 w)	-1.219	648.443	0.223	-0.258	-0.673	0.158
ROM External rotation (12 w)	4.979	623.069	< 0.001	0.926	0.560	1.291

If p < 0.05 it is considered that are difference between the compared series

## Discussions

Although still in evaluation, prosthetic replacement after femoral neck fractures is accepted as optimal treatment in elderly patients and there also studies which showed excellent results of THA in patients with OA. THA is the treatment choice for advanced OA however some assume that the outcomes for THA after FNF are inferior. There is clear that THA is the treatment choice for femoral neck fractures in active elderly patients. Looking up in the literature, we found very few studies which compared THA used for these two groups of patients.

The patient characteristics may explain the higher risk of a poor outcome for the patients undergoing hip fracture surgery and the physiological processes associated with hip fracture (the acute inflammatory stress, hypercoagulable and catabolic states) may account for the increased risk and can lead to perioperative morbidity and mortality. The FNF group were older and had more associated pathologies as the OA group. [6, 7]

In our study, both groups of patients, acute and chronic, have the same preoperative morbidities as diabetes, high blood pressure, heart disease, and the surgical risks were low and the same. Obesity was higher in FNF patients, as well as this group was dominated by the female patients. Even the hip fracture may be associated with physiologic processes that are not present in circumstances leading to elective THR, the risk of morbidity and mortality was the same. We did not have information on the mechanism of injury (fall due to instability, orthostatic hypotension, or poor nutritional status) or other concomitant injuries which may influence the outcome. In our study, the patients in both groups had similar disease severity and experienced similar complication rates. The more comorbidities a patient has prior to the surgery, the higher their risk of a major complication after surgery. We didn’t find any data to suggest that factors intrinsic to hip fracture may influence negatively the outcomes.

Gjertsen et al. reviewed the results of THA done primary after FNF or secondary after failure of osteosynthesis for FNF (8577 cases) and compared to the THA done for primary coxarthrosis (551,090 cases). All patients had cemented arthroplasty and at five years the prosthesis survival was 95.1% in the FNF group and 97.1% in the OA group. The causes for revision in the acute group included pain, deep infection, periprosthetic fracture, dislocation, and femoral component loosening. [8, 9]

We did not have information about the type of the accident in case of hip fractures as orthostatic hypotension, fall due instability or poor nutritional status and probably these unmeasured comorbidities may influence the outcomes. Even if the data from meta-analysis of prospective studies establish that patients with hip fractures had higher risk of mortality compared with the OA patients, these data do not determine if the risk relates to surgery a related to the hip fracture directly. Opposite there are studies who reported unadjusted results. It is obvious that the more comorbidity a patient has prior to surgery, the higher their risk of a major complication after surgery. Sassoon et al. found that rates of in hospital mortality, length of hospital days, pulmonary embolism, hematomas, infections, and dislocations were significantly higher in the immediate perioperative period for the fracture cohort. [10–12]

A hip fractures results in trauma, pain, bleeding, and bed immobility which initiates inflammatory process, hypercoagulability and catabolic stress and lead to complications after surgery. A rapid surgery can minimize these factors and limit the consequences. [13]

Elderly patients with FNF typically have a limited level of activity, which improves prosthetic longevity, but the same patients have worse bone quality that may compromise implant fixation. A limitation of our study was the use of Harris score, Womac score and VAS which are physician-generated outcome measures rather than patient-generated outcome measures and they are likely prone to less interrater reliability. Our results were not stratified by age even we introduced all the patients older than 45 years. [14]

The use of Hardinge approach likely contributed to a low rate of dislocations in both group of patients. It was established in anterior studies that the modified lateral approach of Hardinge has a lower rate of dislocation compared with other surgical approaches. THA after FNF were associated with a higher dislocation rate and limb lengthening due to increased ligament laxity compared with patients with OA. [15, 16] A small number of patients had a leg discrepancy (1.71% vs 2.37%) with not statistically difference between groups and that affected insignificantly Harris hip score and patient satisfaction. The functional recovery showed a slightly improvement for the OA group of patients in terms of Harris hip and Womac score. Our investigation also showed no difference in terms of range of motion (internal rotation, external rotation) between the two groups.

Even when there are selected the healthiest patients, THA for hip fractures is associated with increased risk of mortality and serious complications compared with patients who undergo THA for coxarthrosis. Parvizi et al. retrospectively reported on several patients undergoing hip replacement for FNF and documented a similar 30-day mortality rate of 2.4%. Risk factors for higher mortality after THA for hip fractures included patients who received cemented prosthesis, female, older than 70 years, cardiorespiratory disease. [17]

Le Manach et al. conducted a large review of French National Hospital Discharge Database comparing all patients undergoing hip fracture surgery to elective THA patients. As expected, hip fractures patients were older and had a higher comorbidity burden compared with elective THA patients. Patients undergoing hip fracture surgery were nearly six times more likely to die in the hospital and two and a half times more likely to experience major impatient complication. [1, 18]

Qin et al. suggest that major complications, longer length of postsurgical stay, non-home discharge and unplanned readmission may constitute the increased cost of care for THA after hip fracture. [19]

Abboud et al. found no difference in outcomes for patients undergoing THA for FNF versus those undergoing the same operation for OA. Harris hip scores, preoperative morbidity and mortality were equivalent for both groups. [15] Lombardi et al. found no dislocation in either group, a small number of patients had a leg length discrepancy and a Harris hip score with no difference between groups. [20]

Recent advances in THA resulted in a significantly shorter hospitalization period and a lower complication rate for both groups. [21] Our findings suggest that major complications and readmission to the hospital may constitute the increased cost of care for THA for FNF or OA, but we found no significant statistically differences between the two groups. The retrospective study we conducted showed no difference in outcomes for patients undergoing THA for FNF versus those undergoing the same procedure for OA. Preoperative comorbidities and complications related to the surgery were equivalent for both groups. Functional outcomes were slightly improved for the selective group with OA. Both groups showed a significant improvement of clinical and functional scores (Harris, WOMAC and VAS).

The most important limitation of this investigation is the short duration of the study period and of the follow-up. However, this is one of the fews reports that com- pares baseline characteristics and outcomes in terms of clinical and functional scores in patients with THA for different causes. More studies should examine various time intervals, including 90 days and more than one year after surgery. These data should be used to develop more robust and granular risk-stratification methods that might be applied to prospective payments in alternative reimbursement models.

## Conclusions

Our study showed that THA for treatment of an acute fracture of the femoral neck in an elderly patient can provide results comparable to those of patients who received THA for OA and we found that the results are similar.

## Data Availability

All the data and materials are available upon requests from the corresponding author.
